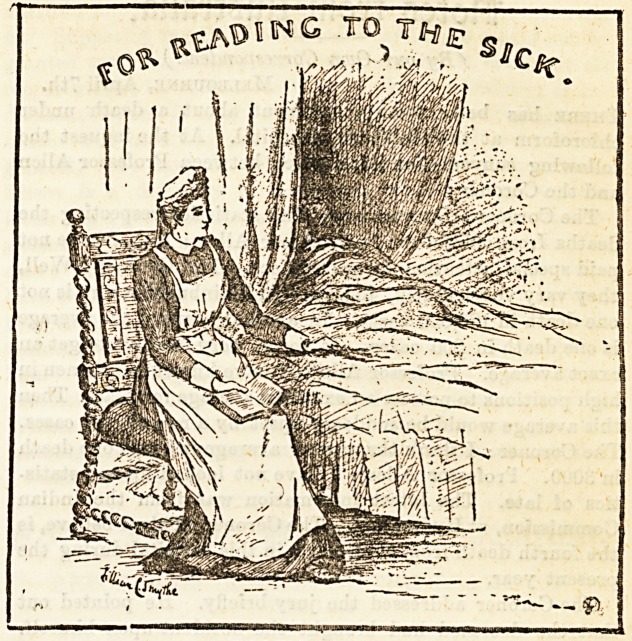# The Hospital Nursing Supplement

**Published:** 1891-05-23

**Authors:** 


### "The Hospital" Nursing Mirror.

Being the Extra Nursing Supplement of "The Hospital" Newspaper.

Contributions for this Supplement should be addressed to the Editor, The Hospital, 140, Strand, London, W.C., and should have the word "Nursing" plainly written in left-hand top corner of the envelope.

### En Passant.

LINCOLN INSTITUTION FOR NURSES.—This slim red report reaches us once more telling of another year passed and over—a year of good work so far as the Institution is concerned. Unfortunately, the work is almost beyond the workers; many cases have had to be refused, and many nurses have suffered from the strain incident upon them. The Institution meditates raising its fees for private nurses, and we hope this intention will be carried out. As a rule one and a-half guineas a week for medical cases, and two guineas for surgical, can, and should be paid.

SALFORD WORKHOUSE.—The report from the Commissioners in Lunacy was read at the late meeting of the Salford Board of Guardians. It stated that Mr. Phillips, one of the Commissioners, had attended at the workhouse in March last, and found in the imbecile wards 79 males, and 118 females. After examination he considered that, with one exception, these ought not to be removed. They were all properly clothed and clean, and the women's appearance, especially, was very creditable to the nurses. He would be glad to see properly warmed and more convenient padded rooms, instead of the present so used mere slices of small kitchens with useless grates in them. In the strong rooms there were many facilities for suicide, and the doors were very slight. The hot-water arrangements were also imperfect. The nursing seemed to be good. Mr. Phillips also made several suggestions for the additional comfort of the inmates.

A STORM IN A TEA-CUP.—The capital of the Highlands is exercised over a small row at the Infirmary, and the story is this: A medical manager in leaving a ward one afternoon met the Matron at the door, and said to her—"Is that girl" (meaning Mary Chisholm) "still making complaints?" adding, "She says she found feathers in the food one day." This conversation was overheard by a patient, a girl, who, being a very nervous person, got greatly excited, fancying the remarks of the doctor to be meant as applying to her. Miss Falconer (the Matron) soothed this girl, and assured her that the remarks did not apply to her, and having said this she turned to Mary Chisholm, and said—"Was it you, Mary, that was making complaints?" Mary replied that she never complained about the food, but that she told her sister that she had been two days before she had been attended to by Dr. Macritchie. Miss Falconer said, "Was it not unkind, Mary, to make such a complaint, as you knew the cause—the want of 'shot' to complete the operation?" Mary said, "I did not tell my sister in the way of a complaint, and I did not mean her to tell anyone, but it is just her way to go off and tell, and I will give her a scolding about it when she comes next time." Miss Falconer then said, "If she behaves in that way it may come to be a question whether she should be allowed to come back." This last sentence of Miss Falconer's causes the warfare; it is called a "threat," and in return for it some people seem to be inclined to hurl "threats" at Miss Falconer. It has been our privilege to see the Matron of the Inverness Infirmary at work amongst her patients, and her gentleness and skill were both remarkable; seldom have we seen a woman more loveable and more beloved. That her careless words were unwise Miss Falconer has acknowledged, and here, surely, this storm might be allowed to end. In our opinion the nursing at the Inverness Infirmary is excellent.

SHORT ITEMS.—The Nursing Institutions have been refusing from 20 to 50 cases a day during the influenza epidemic.—Miss Amy Fowler, who was for a few months notorious under the name of "Sister Rose Gertrude," has married Dr. Lutz, a German savant and agnostic.—A large number of nurses at the London and other hospitals are down with influenza.—Madame Albani sang at Windsor last week in aid of Princess Christian's District Nurses.—Miss Jolliffe, late nurse at the London Hospital, has started a private home for invalids at 52, Harold Road, Cliftonville, Margate.—We would call the attention of our readers to the fact that we have had to repeat queries 3 and 8 this week, as no answers have been received to them.

THE NURSES' CO-OPERATION.—A Scotch paper in an article on this new union amongst nurses says: "Justice to the individual is in theory, at least, dear to every Britain-born heart. Under its banner we are each and all of us ready to step out, and do doughty deeds. It is, therefore, with confidence that we bespeak your good offices in laying—through the pages of the *Scottish Pulpit*—before the public a subject meet for the consideration of all among them, seeing that it is not to the few but to the many that sickness, and its woeful train of divers distresses, come. And when it *does* come, do we not all know, and heartily concede, that it is to those 'ministering women' who answer so promptly to our call for aid, much honour and great gratitude is due? That being so, it goes without a great deal of saying that we are willing to take a step further and make our gratitude tangible by putting *our* shoulders, too, to that wheel which has been fairly set in motion to promote a scheme recently formulated, its object being briefly as follows: 'To supply private nurses, thoroughly trained and certificated, to practitioners and the public, through the agency of nurses who have combined for their mutual benefit and welfare.'" After this we think the day cannot be far distant when a branch of the Co-operation will be established in Glasgow.

WORKHOUSE INFIRMARY NURSING ASSOCIATION.—On May 14th the Duchess of Teck presented the medals won by the Mary Adelaide nurses during the year. The ceremony took place at the Nineteenth Century Art Galleries, and was a very pretty one. Nearly all the nurses carried big bunches of flowers, and the Duchess and her daughter were most gracious and kind to all. The following received medals: Miss Jane Court, Emily Tucker, K. E. Richmond, Helen Ogg, Annie Brown, J. A. Bower, Clara Smith, L. Dancket, E. Sanford, Ada Fox, Jane Lewis, and M. Guernsey. The following were not able to be present, so medals were sent to them: A. G. Brennen, Elizabeth Hardy, J. Hodgetts, Emeline Dodson, M. G. Willard, Lissie Allen, Alice Parnell, Marion Watkins, and Angelina Millage. Gratuities ranging from £1 to £2 were awarded to 22 nurses for long and faithful service. When the presentations were over the Duchess took a seat in the centre of the hall and heartily enjoyed a very clever conjuring entertainment that followed. Amongst those present were Lady Knutsford, the Hon. Mrs. J. G. Talbot, Miss C. J. Wood, Miss Wilson, Miss Rosalind Paget wearing her Queen's Badge, Miss Gibson from Birmingham, and many others. The association is in need of both nurses and probationers just now, and those anxious to learn nursing should apply at the office, 6, Adam Street, Strand, any morning between eleven and one.

### Lectures on Surgical Ward Work and Nursing.

By Alexander Miles, M.B. (Edin.), C.M., F.R.C.S.E.

### Lecture XXV.—THE LONG SPLINT

Uses.—This splint, which is associated with the name of Liston, who introduced it, is largely used in the treatment of fractures of the femur, whether of the neck or shaft. It controls the whole limb and the movements of the trunk on the thigh at the hip joint, thus securing absolute rest to the injured part. It may be used single or double, *i.e.*, one on each leg, and it is often used as an addition to other splints; for example, in fracture of the shaft of the femur, the break in the bone is first controlled by means of short gooch splints, and then the whole limb by a long splint. The double long splint is used chiefly in children, in whom the difficulty of keeping them from wriggling about is so great. It also facilitates the cleansing of the child. Some adults are not less easily kept quiet than children, and in such a double long spint is of great value.

(1.) Single Long Splint.—There are two methods of employing this splint; the old way, in which the extension is applied by means of a peroneal band, and the more modern, and much preferable, way, in which the ordinary apparatus with weight and pulley affords the necessary extension. (a) Old Method: Materials required—(1) Long splint, with rest for it, (2) sheet, (3) wool for padding, (4) several long strong pins (cap pins), (5) broad domette bandage, (6) two large handkerchiefs, (7) safety pins.

Method of Preparing Materials.—The splint should be about four inches broad, and should extend from the axilla to an inch or two beyond the foot. About two inches from the upper or axillary end two holes about the size of a shilling piece are made two inches apart, while at the lower end two triangular wedge-shaped pieces are cut out. Lay the splint alongside the patient, with the upper end well up in the axilla, and mark on it with a pencil the level of the great trochanter at the upper end of the thigh, and of the malleoli at the lower end. Now fold your sheet to this width, and roll it carefully round the splint, covering, of course, only the part between your two pencil marks, and leave as much of the sheet unwound as will encircle the limb and leave a margin with which to fix the apparatus. To compensate for the greater circumference of the leg at its upper part the sheet should be folded slightly obliquely. It is sometimes desirable that the sheet should pass from the splint over the front of the limb, and then encircle it, sometimes over the back of the limb. This depends on whether eversion or inversion has to be corrected, and will be decided by the surgeon, and you will have to fold the sheet accordingly. Having enclosed the splint in the sheet, carefully pad the upper part which comes into contact with the side of the chest. This is best done with evenly cut pads of wool tied on to the splint with pieces of bandage. Leave the holes free. The leg should be thoroughly washed with soap and water, and dusted with a mixture of powdered boracic acid and starch, and then all bony prominences carefully padded with antiseptic wool. All being ready the free part of the sheet is passed under (sometimes over) the limb, which is carefully steadied and held in the proper position by a competent assistant, and having been brought round is fixed to the part enclosing the splint by means of long pins. The foot is fixed to the lower end of the splint by passing one of the handkerchiefs as a clove hitch round the ankle, and then tying the ends through the notches at the foot of the splint. Now by gently pushing on the upper end of the splint, in its long axis, the leg is drawn upon and extended. How is this extension to be kept up? By means of the other handkerchief—the peroneal band. This is passed under the peroneum and brought through through the two holes at the top of the splint, passing them from within outward. Now by pulling on these ends the splint is drawn down and keeps up the extension on the foot. I have described this method fully because it is very useful, especially in district and private nursing practice, where on account of the shape and arrangement of beds, and from other reasons, the extension apparatus is unavailable. When it is possible, however, it is advisable to use the extension apparatus.

Disadvantages of the Peroneal Band Method.—(1) The handkerchiefs gradually yield to the strain, elongate, and so the extending action is lost, and the constant readjustment interferes with the absolute rest which is so essential to the successful treatment of these cases. (2) The parts to which the handkerchiefs are attached are constantly being chafed, and soon get excoriated, adding additional inconvenience to the patient. (3) In spite of all precautions, the peroneal band gets soiled, especially in children, and the necessity for changing it interferes considerably with its usefulness.

Double Long Splint.—This is simply the application of a single long splint to each leg, the lower ends of the two being fixed into a common foot-piece. The advantage of thus fixing both legs is that it prevents all movement, and, in children especially, facilitates cleanliness.

Vertical extension is another method of treating fracture of the thigh in children. This consists simply in handing the child up by the feet with the limbs at right angles with the trunk. Extension plasters are applied to each limb and the tapes attached to a cross-bar over the bed. This is of great assistance in keeping children quiet as well as clean and dry.

(*b*) Long Splint with Extension Apparatus.—Materials required: Same as for the old method, and, in addition, all the apparatus needed for extension (page xx). Method of application: The extension being applied as before directed, the splint is prepared and adjusted in exactly the same way as already stated, save that the peroneal band is omitted. The foot of the bed must be raised to obtain the necessary counter extension. Advantages of this method: The extension is constant; there is little chance of the apparatus being soiled, and no danger of excoriation if proper precautions be taken to pad all prominences; thus, altogether, it is easier to insure the maintenance of absolute rest, which is the secret to rapid and accurate union.

### Death in Our Ranks.

We hear with regret of the death of Miss A. M. Knight, M.B., at Vienna, at the early age of 26. Born in South Australia, she passed the matriculation examination of the University of London at Adelaide, where she attended the University and carried off the Sir Thomas Elder prize for physiology in 1883. In the spring of 1885 she came to England and entered at the London School of Medicine for Women and the Royal Free Hospital, where she pursued a most diligent course of study and obtained many distinctions. After passing the M.B. Lond. Examinations, in November, 1889, with honours, she was received at Marlborough House by the Princess of Wales, being the first Australian lady who had received the degree in medicine of the University of London. Immediately after taking her degree she was appointed resident medical officer at the New Hospital for Women, and in that capacity carried out the alterations consequent on the removal of this institution from Marylebone Road to larger premises in the Euston Road. Last June the trustees of the Helen Prideaux prize unanimously conferred it upon her, this prize being the highest distinction connected with the London School of Medicine for Women. In accordance with the terms of the award, she proceeded for further study to Vienna, where she died, after a short illness, of typhlitis.

### A First Day.

MY dear Ircree,—You ask if I have really made up my mind to be a nurse, and if so, why in a fever hospital? My answer to the latter question is, because I am too young to enter into a good general hospital; the age at which probationers are taken usually is twenty-five. To the former, I reply yes, and it would be well if you, too, were to give up the aimless life which we have led since leaving school, and which I know you are fond of still. You cannot tell how delightful it is to feel that you are doing some little good in the world, and though hospital life is by no means all honey, I do not think I should care to give it up. Sometimes I look back upon last year, thinking of the many pleasant days spent with friends in riding, boating, etc., of the merry parties or concerts I attended in the evenings, then upon this year, here, where I am only "that probationer," and must be taught her place as such, if she is so presumptious as to speak to the nurses, and wonder if I regret that I decided in haste to be a nurse. As yet I cannot tell. I give you an account of my advent and first day here. Not having a long journey, I arrived at the town nearest my destination about one o'clock. Soon I was packed into a cab, and after being jolted and tilted out of my place several times, stopped before a large entrance gate. The driver put his head through the window, saying "which part, miss?" I said "Nurses' Home," and the cab rattled on to a door, at which stood a neat maid. "Is that the new probationer?" I heard her ask, then she disappeared, and I was left standing in a long corridor, neither person nor door to be seen. I was feeling very uncomfortable, when I saw a nurse coming, who, as she took my hand, said, "My dear, you are just in time for dinner; the Matron is away. I will take you to your room and give you a cap and apron, then you will come down to dinner." The apron was soon put on, but the cap I could not "settle." The maid coming in to take me downstairs, I made one last desperate effort to get it on, and as I took a final glance in the huge mirror thought it looked, on my curly half-short hair, as if it had been stuck on a broom. Soon I was seated at table. The nurse I had seen was the night Superintendent, doing duty for the Matron; she was addressed as Sister. After dinner she asked me to change my dress. I stayed in my room for the rest of the day, the maid bringing me some tea at six. My bedroom was large and well furnished, but had no fire-grate, and only a small piece of carpet by the bed.

"Have you seen the new pro.?" I frequently heard, as foot-steps passed up and downstairs, my room being the first on the landing. The answer was generally "no": but once, "yes, and she looks as if she was to be nursed, instead of nurse, so pale and delicate, I am sure she will take something."

Next morning at seven I was sent to the enteric wards; my work was to clean the taps, trays, anything bright that I saw, sweep, dust, keep up nine fires, the coals having to be carried from the corridor; I did not think I could carry the cool, but I soon found I could manage very well. The same morning I was vaccinated. At dinner I saw two day Sisters, who wore dark print dresses and pretty caps, some twenty-five nurses, looking all very much alike in light print dresses and plain caps. After dinner I was "off duty" until half-past five. I had tea in the dining-room at five, then went back to the ward, where everybody soon began to be busy, and I in their way, until nine o'clock the night nurses came on, we day people went to supper and prayers, then to the sitting-room. One nurse was killing "Santa Lucia" on the piano, some were talking, some reading. One girl asked me if I would go out; so we walked round the grounds—at least, all but the small-pox buildings, near which I was afraid to venture. At half past ten we went to our rooms, and I, being very tired, was soon asleep.

SPRING TIME.

THE winter has at last gone, and spring, which had seemed to linger unduly, has burst upon us in all its beauty. The flowers have been struggling to show their tender heads for weeks, but the sharp winds have nipped them in the bud or shorn them of half their charms. No sooner, however, do the soft, warm showers fall, and the sun shine forth in its glory, than with one accord the hedges become green, the trees start into sudden life, and one and all rejoice in renewed vigour of leaf and blossom, and join the birds in giving praise for gifts so lavishly bestowed.

We cannot account for either the way in which God sends these gifts or holds them back at His pleasure, for "He moves in a mysterious way, His wonders to perform," so that what has seemed to us a time of trial may have only been used by Him in preparing for a season of more than usual fruitfulness.

The earth is not perfect as when the great Creator looked on the finished works of His hands, and pronounced them "very good." Since then sin has crept in, and with it thorns and thistles to disfigure and spoil the land. Probably the severe winter and the unusual drought have been sent as wise correctives.

Nature will not have us watch her in her working; she veils all she does in silence.

"Who ever saw the earliest rose
First open her soft breast?"

says the Christian poet, Keble. You may long fix your gaze on it, and can trace no change;

"But look away a little space,
Then turn, and lo! 'tis there."

As with the world of nature, so with the world of grace. We do not see God working in our hearts, and fancy He cannot be there because He allows trials, sickness, unkindness of those we love, and the cold blasts of adversity to blow upon us, and apparently check or kill our happiness, while these things have really been sent to save us from ourselves, to keep down our pride, our love of being first or our indolence, from, in a word, ruining the body and soul. He keeps us low, dry, sad, weary, but having brought us to our knees He pours on us the dew of His love and the soft showers of the Holy Spirit; we become responsive to His gifts, and when the sun of Righteousness shines into our hearts we blossom and bear fruit to His glory. Let us then take all things, whether of nature or of grace as sent by One who doeth all things well. If we do not see His finger we know it is there pointing and directing our way to Him. Let love to Him grow silently in our hearts strike its roots deeply there, seen only by God and His good angels. It cannot be hid for ever, it will, without fail, spread its fair blossoms on all around in charity and good works and men will say "whence come these lovely flowers and fruit? See these Christians how they love one another."

### Notes from Australia.

*(By our Own Correspondent.)*

Melbourne, April 7th.

There has been some excitement about a death under chloroform at the Melbourne Hospital. At the inquest the following conversation took place between Professor Allen and the Coroner:

The Coroner: Do you know the statistics respecting the deaths from chloroform? Professor Allen: No; I have not paid special attention to the subject. The Coroner: Well, they vary very greatly, indeed. In Edinburgh there is not one death in 126,000 cases. In another hospital the average is one death in 200 cases. It seems very difficult to get an exact average. Professor Allen: I have known gentlemen in high positions to possess an excellent average for years. Then this average would be suddenly upset by a run of bad cases. The Coroner: I think the proper average is about one death in 3000. Professor Allen: I have not looked up any statistics of late. The latest information was from the Indian Commission, at Hyderabad. The Coroner: This, I believe, is the fourth death from chloroform in this hospital during the present year.

The Coroner addressed the jury briefly. He pointed out that the deceased had brought the accident upon himself. The injuries sustained necessitated an operation. There was always a risk in giving chloroform in such cases. The man, however, would have died in any case in twenty-four hours. The evidence of Dr. Molloy, which was to be relied upon, showed that Dr. Craig had administered the chloroform carefully and properly. Professor Allen, whose opinion was valuable, was of opinion that an expert in giving chloroform should be appointed to the hospital. All they had to be satisfied of was that the chloroform was given properly. It might take years, but it was certain that a proper chloroformist would be appointed to the Melbourne Hospital. The jury found that the deceased died through suffocation by chloroform, and that the chloroform was carefully administered by Dr. Craig.

The Committee of the Women's Hospital have adopted a resolution to the effect that a communication should be sent to all the Melbourne hospitals, inquiring whether they would be willing to appoint sub-committees to confer with the Committee of the Women's Hospital with a view to securing more uniform arrangements and rules for the training of nurses. At the same Committee was read an interesting letter received from Mrs. Ormond giving a description of European hospitals, and especially the new Victoria Hospital at Glasgow, which she has recently visited. Mrs. Ormond detailed the system of ventilation in the hospital. In hot weather air is pumped through a screen of hemp cords placed at an angle of 45 deg., over which water is discharged at intervals, and is then driven by a fan through the ward. In cold weather the air is heated by being passed over hot tubes after being purified by being driven over the damp hemp. Mrs. Ormond also stated that moveable electric lights above each bed are found very convenient in the home hospitals, and that the walls in the wards are partially tiled with white glazed tiles.

At the last meeting of the Charity Organisation Society Mr. J. Goldstein, for the information of the Council, related an experiment which had been tried recently. He learned on the 11th inst. that eight men could find work at a place in the country about two hours' journey from Melbourne, at pay which, by division of labour, would enable them to earn 15s. or £1 a week each beyond their keep. The preliminary expenses were kindly guaranteed by Professor Morris. Of five men who were offered the work as an experiment, one pleaded that he was in delicate health and could not sleep in a tent, although he had never mentioned the fact before; the second alleged heart disease, also the first mention of such a malady; a third stated that the letter had reached him too late; a fourth never called at the office at all, and the only one eager to go was a paralytic, who, when it was pointed out that the weight of the tent and "swag" would be too much for him without companions, nevertheless protested that he was "game to tackle it." He was given the position of "boots" at an hotel, which one of the other men had also refused.

Professor Stuart has returned from Berlin and given an exhaustive statement of the rise, progress, and fall (?) of the Koch consumption cure. The final result of the whole story was not so much disproof of Dr. Koch's statements as disappointment of the hopes of the public. The public, indeed, hoped for far more than Dr. Koch ever promised. In conclusion, Dr. Stuart described the circumstances attending Dr. Koch's being in a manner forced to publish his discovery before he was ready, and stated that no one was more disgusted than Koch himself. The lecturer gave it as his opinion that the remedy was a useful aid in diagnosis, but nothing more.

### Nurse Training in the Seventies.

By E. D., Author of "Recollections of a Nurse."

*(Continued from page xl.)*

The Sister of this medical ward did not possess that quality which Saint Francis of Assisi calls courtesy, and I was truly thankful after a week or two to be sent into the women's surgical ward, under a Sister who was good, clever, and beautiful to look upon. Here I was for seven months straight off, perfectly happy, notwithstanding very hard work and sore throats, and being assistant nurse I had less washing of basins and cups, but there were bright polished bowls to be cleaned twice a day; they were used to receive the dressings taken off surgical cases, and all surgical nurses vied with each other in the matter of bright bowls; but there was also the delightful feeling of watching the wounds healing, some as if by magic. Then the busy operation days. I remember so well my first going to the operating theatre. A man had a tumour taken from the upper part of his arm, weighing 4 lbs. 7 oz. It was the first time I had seen a patient put under chloroform, and I watched most intensely; it is such an awesome thing to see consciousness go in that way, to find before you the stillness of death, then the quiet look of the operator, who seems to take in everything and everybody at a glance—all are ready, all in their appointed places. Then comes the thin red line; if you lift your eyes you see rows of eager-faced students, who watch every turn of the operator's hand—but I have to wash sponges and pass them to the theatre nurse. Still, I manage to see somethingby bending my head first one side, then the other. Sister Annie had taken the precaution to pin my cap all round, knowing my propensity for seeing everything, but she had forgotten to tell me that when the patient was taken out of the theatre I should go too. So I took my stand beside the great operator and he began his lecture. I almost saw the words coming out of his mouth. When the lecture came to an end another patient was brought in, and I went back to my ward. It was whilst assistant nurse in this ward that a patient to whom I was about to give a bath informed me that forty-and-seven years she had been in the world and had never had such a thing—"Never had such a thing!" repeated the irate patient. "You don't suppose that all patients who come to the hospital are dirty, do you?" However, I prevailed upon her to begin her forty-eighth year with soap and water. About this time I had a bad throat, and was warded, and after my throat got well I was allowed to sleep in the hospital. This meant better breakfasts and suppers, though a little longer time on duty. Here we had bacon or fish for breakfast, sometimes marmalade, and for supper meat and pudding, of which you could have both. But you were on duty from 8 a.m. until a quarter to 10 p.m. At 11 a.m. you generally spent five minutes eating some bread and cheese; beer you could have if you liked. You had twenty minutes to eat your dinner in, you had half an hour for your tea, and the same time for your supper between eight and nine o'clock. Every other day, as I said before, you had two hours off; beyond this a probationer or assistant nurse hardly ever sat down, and this went on every day alike for twelve months at a stretch. When I had been there twelve months and was going for my holiday, the Sister said: "What are you going to do?" "Buy a water pillow and a few extra nightgowns," I answered, for truly the weariness was great. Returning from my holiday I was put on night duty. Here all is different. Your work begins at a quarter to 10 p.m., only half the number of nurses being required for the same number of patients. At midnight we had a penny loaf, about an ounce of butter, an egg, or slice of ham, with tea or coffee. You could eat it all at once, or divide it and have the other half at 4 a.m. At 5.30 the patients had their breakfast—eggs, bread and butter, tea, milk, or cocoa. This was all cleared away by a quarter-past six; then you make the beds and work as hard as you can. Twenty-four beds to make—some of them helpless patients—is no easy matter for two women, considering how often one or the other may be called away, or in the case of a patient who needs constant watching the bedmaking may devolve upon one. Then you must have your night's report ready and the wards dusted by a quarter to eight, to give over to the day nurses. You change your gown and go to chapel, then to the dining-room; there you find hot roast mutton or beef with vegetables, and hot coffee. This is the night nurse's principal meal; after this meal the night probationer of every ward takes the bread and cheese and beer up to the day nurses for their lunch. All the people who have been on night duty can then go out until twelve o'clock. Then you go to bed; the dressing bell rings at 8 p.m. Prayer follows, and supper, generally of meat cooked up and milk pudding; you could have beer or porter, tea or cocoa. You carry your penny loaf, &c., up with you, and so the night's work begins again.

### Everybody's Opinion.

### MALE NURSES.

"ONE OF THEM" writes: In referring to the article on "Male Nurses" your correspondent "M" evidently thinks the writer thereof undervalues asylum training. Nothing of the kind! He thinks asylum training a very valuable acquirement in male nurses, and probably in discussing the question it is one of the points worthy of consideration, whether a period of asylum training is not essential in any definite system of training for a certificate. Reading between the lines, his letter implies that some strictly military system of training is being advocated by a military trained male nurse, neither of which is correct. The writer is not a military man; more than that, he has never been inside a military hospital during his life, and purposely avoided going into any detailed system, aiming rather at drawing general attention to what, in his opinion, was a general want in male nursing. Yet it is a well-known fact that amongst our best male nurses there are those that have been trained in the wards of military hospitals. I am afraid I must agree to differ with "M" when he states that what they learn there "is not likely to be much appreciated in civil life." It is of the utmost value. Further than that, I must demur to his statement as to the percentage of special knowledge to be acquired only in asylums. I am glad that "M" agrees with me in thinking a certificate necessary. That very materially narrows any difference of opinion there may exist between us. Supposing it will be admitted a certificate would not be granted until one is fully qualified to receive it, we meet all requirements, for with a certificate a definite training is indispensable, and if they became general those engaging male nurses will naturally see they get certificated men, as, in general, they now do when engaging females as nurses. There is a difficulty in answering Walter Maguire; his letter has a good manly ring in the tone of it, but is not consistent. He first states: "We want no so-called training for men;" then "Half the attendants from public institutions have to be trained afterwards for private nursing;" further than that, he makes this extraordinary statement: "The best trainer a male nurse can have is the medical man who employs him." According to his own showing, training is necessary; that being so, if only in the interests of one's afflicted patients, Walter Maguire, should he not endeavour to get the training first, and engagements as a male nurse afterwards? I think so, at least!

### Royal British Nurses' Association.

### REFUSAL OF LICENSE BY THE BOARD OF TRADE.

As was indeed inevitable from the first, the Board of Trade have refused the application of the Royal British Nurses' Association to register as a limited company without the use of the word "Limited." The Board of Trade have received a large number of communications from bodies of persons whose interest in hospital nursing is unquestionable and whose experience entitles them to speak with authority, strongly objecting to the issue of such a license. After careful consideration of the objects of the Royal British Nurses' Association, and of the representations made in opposition thereto, the Board are unable to satisfy themselves that the means which the Royal British Nurses' Association propose to adopt are either adequate to carry out their object satisfactorily or so free from objection as to warrant the Board of Trade in the issue of a license. Under these circumstances, with a full knowledge of all the facts, the Board of Trade have refused the application of the Royal British Nurses' Association, and have declined to grant a legal recognition to its register or its objects under section 23 of the Act of 1867.

### Presentation.

DOVER NURSES' INSTITUTE.—At Easter the staff of Nurses belonging to the Dover Institute presented Miss Ayliff, the Lady in Charge, with a silver cream jug and sugar basin as a token of their love and respect.

### Notes and Queries.

TO CORRESPONDENTS.—1. Questions or answers may be written on post-cards. 2. Advertisements in disguise are inadmissible. 3. In answering a query please quote the number. 4. A private answer can only be sent in urgent cases, and then a stamped addressed envelope must be enclosed. 5. Every communication must be accompanied by the writer's full name and address, not necessarily for publication. 6. Correspondents are requested to help their fellow nurses by answering such queries as they can.

### Queries.

(11) *Stains.*—Is there any method of removing stains from water-beds?—*Inquirer.*

(12) *Smell of Sulphur.*—How can I get rid of the smell of sulphur from a feather bed and pillows fumigated for disinfection?—*H.*

### Answers.

*Beta.*—The clinical slate is supplied by Messrs. Fannin and Co., 41, Grafton Street, Dublin.

*C. J.*—For information concerning Lady Dufferin's Fund, apply to Colonel J. Robertson, 27, Inverness Terrace, Hyde Park, W.

*Nurse E.*—Get "A Guide to District Nurses," by Mrs. Dacre Craven. Published by Macmillan. Price 2s. 6d.

*J. de L.*—We have no room to print the lines sent.

*A. Moore.*—The screen has been photographed by Messrs. Elliott and Fry, 55, Baker Street, W. We will give further particulars in a week or two.

*Dorothy.*—You know best whether you prefer mental work; if so, try and get some training. Apply to the Matron, Berrywood Asylum, Northampton.

*Miss Borradaile.*—Your letter will appear next week.

### Human Flowers.

It was a poor man's garden, the little home, and its uses were of the practical order. But it had one flower—a Daisy. She was a small, white-cheeked mite, with a spiritual look in the clear, round eyes. The simple-minded parents regarded, with secret awe, the solemn little toddler whose golden head did not nearly reach up to the green bushes, in and out of which she threaded her way through the livelong days of summer.

"Her don't, somehow, seem to belong to we; her's like chaney, so fine and brittle-like, for all the world same as gentry's children, Susan."

"Perhaps her's only lent to we for a bit, John," whispered back the fearful mother with the meek brown eyes, the calm, untroubled eyes one never meets in city streets. But Daisy was mortal enough in one respect: everything the mites of pink hands could grasp went straight into the button of a mouth. The brighter the object the nicer it must taste; therefore one still, sunshiny day a handful of crimson berries was a prize. Then the sun's light was darkened; a tempest swept over the humble little garden. Its one flower, its pride, drooped and fell to the earth. The poison of the gleaming berries had begun its deadly work.

"I knowed it," wailed the frantic father, "her wor too dainty for such as we. God will tear her from us," and he beat his breast with useless hands as he hung over the little one's agonies.

But the mother did not speak, only a new born anguish sprang up in the calm brown eyes. Lifting the dying child in her arms, she went silently out of the home; away out into the sweet-scented night. Tramping unwearily on with her burden, over miles of country lanes and fields, she reached at last the distant little town, never halting until she stood at the gates of its modest hospital.

"Save my child!" rang out the hoarse cry; then there was a frozen silence while the mother "waited for the Lord." Would He never "incline unto" her, and "hear her calling"? Long hours, centuries they seemed, dragged on; then the dumb prayers were answered.

"We have saved your child!" and the mother's arms clasped tight her treasure. The storm was over and spent; the Daisy was spared to bloom again, all innocently white in the humble garden.

In the hot-house, where money was lavished on the culture of all that was rare and difficult, there was over-much store set on a human blossom.

"Our boy will grow up, and reign here after us," said the proud father, "and keep up the old name in the land."

"Yes; keep it up as you have done—by great works and enterprises for the good of others," and the pair gazed exultantly at the brave, bold boy who flashed here and there, too quick and alert for anyone to be his keeper. But children are all of one pattern. Like the one flower of the cottage garden, little Guy's treasures were apt to go the same road as Daisy's poison berries.

"I've eatett a key!" was the boy's announcement, calmly made, one rainy day, when he had been given an old casket that he might amuse himself over its contents—and then he began to cough. It was found to be true, and over all the great house consternation flew as the angel of death flapped its wings with startling suddenness. Telegrams sped to town for the help of the highest in the profession.

"Get Sir This and Sir That!" was the frenzied cry, but time was flying, and the boy was choking. Such remedies as were known were, all in vain, essayed.

"Let him be driven off to the cottage hospital," was the suggestion of a practical mind.

"*My* child to the cottage hospital! Never!"

"You are outrageous!" gasped hysterically the mother; "I shall not part from my boy."

"But they must know something, there, that can be tried."

"Silence, I tell you!" broke in the father. "He would be exposed to all sorts of fevers and horrible diseases there. Bring doctors here; all you can find; in mercy bring them!" and he wrung his hands. But country practitioners cannot always be found at a moment's notice. While the country is being scoured for them, the precious moments are flying. All in vain the expostulation; the chance was thrown away.

Presently the brief battle came to an end; the little life that struggled bravely while it could, died out abruptly; and when the train brought the London physicians they were in time to tell what everybody already knew, that little Guy, the choicest flower of the splendid home, was dead, and quite safe from all fear of infection in the shape of fevers and other horrible diseases.

### Appointment.

[It is requested that successful candidates will send a copy of their applications and testimonials, with date of election, to THE EDITOR, The Lodge, Porchester Square, W.]

BOLTON INFIRMARY AND DISPENSARY.—Miss Jane Anstey has been appointed Matron of this Infirmary, and commenced her duties on the 20th inst. Miss Anstey trained at St. Thomas's, and for the last eight years has acted as Matron of the Manchester Southern Hospital, on leaving which she was presented by the nurses and servants with handsome presents.

### Amusements and Relaxation.

### SPECIAL NOTICE TO CORRESPONDENTS.

**Second Quarterly Word Competition commenced April 4th, ends June 27th, 1891.**

Competitors can enter for *all* quarterly competitions, but no competitor can take more than *one first prize* or *two* prizes of any kind during the year.

Proper names, abbreviations, foreign words, words of less than four letters, and repetitions are barred; plurals, and past and present participles of verbs, are allowed. Nuttall's *Standard* dictionary only to be used.

N.B.—Word dissections must be sent in WEEKLY not later than the first post on Thursday to the *Prize Editor*, 140, Strand, W.C., arranged alphabetically, with correct total affixed.

The words for dissection for this, the EIGHTH week of the quarter, being

"RAINBOW."

| Names. | May 14th. | Totals. | Names. | May 14th. | Totals. |
|---|---|---|---|---|---|
| Christie | 40 | 216 | Ivanhoe | 40 | 187 |
| Patience | 42 | 214 | Weta | — | — |
| Agamemnon | 40 | 218 | Lady Betty | 35 | 204 |
| Hope | 41 | 220 | Mortal | — | 76 |
| Reldas | 39 | 214 | Little Eliza | — | — |
| Lightowlers | 41 | 208 | Dove | — | 95 |
| Nurse J. S. | 32 | 177 | Ladybird | — | — |
| Qu'appelle | — | — | Psyche | 37 | 185 |
| Jenny Wren | 39 | 202 | Ugug | 37 | 157 |
| Wyameris | 41 | 215 | Harrie | 35 | 53 |
| Paignton | 39 | 186 | Grannie | 41 | 170 |
| Theta | 36 | 206 | Eale | 40 | 169 |
| Success | — | 17 | Grimalkin | — | 53 |
| Tired | — | 136 | Nurse G. P. | 21 | 25 |
| M. G. | 35 | 188 | | | |

**Notice to Correspondents.**

N.B.—All letters referring to this page which do not arrive at **140, Strand, London, W.C.**, by the *first post on Thursdays*, and are not addressed PRIZE EDITOR, will in future be disqualified and disregarded.

## Figures and Tables

**Figure f1:**